# Digital Mental Health Interventions for Alleviating Depression and Anxiety During Psychotherapy Waiting Lists: Systematic Review

**DOI:** 10.2196/56650

**Published:** 2024-09-10

**Authors:** Sijia Huang, Yiyue Wang, Gen Li, Brian J Hall, Thomas J Nyman

**Affiliations:** 1 Faculty of Arts and Sciences New York University Shanghai Shanghai China; 2 Center for Global Health Equity New York University Shanghai Shanghai China; 3 School of Global Public Health New York University New York, NY United States; 4 Health, Behavior and Society Johns Hopkins Bloomberg School of Public Health Baltimore, MD United States; 5 School of Psychology and Clinical Language Sciences University of Reading Reading United Kingdom

**Keywords:** digital health, digital technology, digital intervention, digital interventions, waiting list, digital mental health intervention, DMHI, digital mental health interventions, DMHIs, digital mental health, mental health intervention, mental health interventions, mental health, mental illness, mental disease, mental diseases, mental illnesses, depression, depressed, major depressive disorder, MDD, depressive disorder, depressive, anxiety, anxious, self-guided, self-guidance, self-mediated, self-mediation, systematic review, systematic reviews, mood disorder, therapy, tele-therapy, web-based therapy

## Abstract

**Background:**

Depression and anxiety have become increasingly prevalent across the globe. The rising need for treatment and the lack of clinicians has resulted in prolonged waiting times for patients to receive their first session. Responding to this gap, digital mental health interventions (DMHIs) have been found effective in treating depression and anxiety and are potentially promising pretreatments for patients who are awaiting face-to-face psychotherapy. Nevertheless, whether digital interventions effectively alleviate symptoms for patients on waiting lists for face-to-face psychotherapy remains unclear.

**Objective:**

This review aimed to synthesize the effectiveness of DMHIs for relieving depression and anxiety symptoms of patients on waiting lists for face-to-face therapy. This review also investigated the features, perceived credibility, and usability of DMHIs during waiting times.

**Methods:**

In this systematic review, we searched PubMed, PsycINFO, Cochrane, and Web of Science for research studies investigating the effectiveness of DMHIs in reducing either depression or anxiety symptoms among individuals waiting for face-to-face psychotherapy. The search was conducted in June 2024, and we have included the studies that met the inclusion criteria and were published before June 6, 2024.

**Results:**

Of the 9267 unique records identified, 8 studies met the eligibility criteria and were included in the systematic review. Five studies were randomized controlled trials (RCTs), and 3 studies were not. Among the RCTs, we found that digital interventions reduced depression and anxiety symptoms, but the majority of interventions were not more effective compared to the control groups where participants simply waited or received a self-help book. For the non-RCTs, the interventions also reduced symptoms, but without control groups, the interpretation of the findings is limited. Finally, participants in the included studies perceived the digital interventions to be credible and useful, but high dropout rates raised concerns about treatment adherence.

**Conclusions:**

Due to the lack of effective interventions among the reviewed studies, especially among the RCTs, our results suggest that waiting list DMHIs are not more effective compared to simply waiting or using a self-help book. However, more high-quality RCTs with larger sample sizes are warranted in order to draw a more robust conclusion. Additionally, as this review revealed concerns regarding the high dropout rate in digital interventions, future studies could perhaps adopt more personalized and human-centered functions in interventions to increase user engagement, with the potential to increase treatment adherence and effectiveness.

## Introduction

Poor mental health is an increasing global challenge [1,2], with the prevalence of depression and anxiety reaching a peak during the COVID-19 pandemic [3-5]. Several meta-analyses have concluded that psychotherapies, such as cognitive behavioral therapy (CBT), acceptance and commitment therapy, and problem-solving therapy (PST), are as efficacious at treating depression and anxiety as pharmacotherapy, and that their effects are more enduring [6-8]. Compared to pharmacotherapy, psychotherapy is often the preferred treatment for depression and anxiety by most patients, as well as clinicians [8]. According to the latest Mental Health Gap Action Program guideline by the World Health Organization [9], psychotherapy, instead of medication, is usually recommended as a frontline treatment for common mental disorders.

However, access to psychotherapy treatment remains limited worldwide, even in high-income countries [1]. The insufficient number of clinicians has led to prolonged waiting times before face-to-face psychotherapy can begin. For example, in Germany, waiting times are on average 12.5 weeks for an initial consultation and 23.4 weeks for the first therapy session [10]. In the Netherlands, patients wait at least 6 weeks for the first treatment session [11]. In Hong Kong, while urgent and semiurgent cases wait for 1-4 weeks, stable cases, which are 76% of the total cases, wait for an average of 40 weeks [12]. A prolonged time spent on waiting lists for therapy can have negative impacts on the well-being of patients, demonstrated by increased symptoms of depression and anxiety, remission, deterioration in life quality, and even an increase in mortality [10,13-16]. This delay is also significant for most patients as their symptoms do not usually dissipate naturally with time [10,14].

Although waiting list interventions (ie, interventions that are implemented during the weeks or months before face-to-face treatment) are potentially important for patients who are waiting for treatment, few studies have investigated their impact on reducing depression and anxiety. However, Grünzig et al [17] conducted a systematic review of low-intensity interventions to reduce depressive symptoms before outpatient psychotherapy and found limited evidence of their effectiveness in reducing depressive symptoms. Among the reviewed studies, the interventions were a mix of face-to-face sessions, web-based training for self-help strategies, and supervised bibliotherapy with feedback via email or telephone [17]. Due to the wide range of intervention formats, Grünzig et al [17] also found that acceptance varied between the interventions.

Responding to this treatment gap, digital mental health interventions (DMHIs) are potentially a solution to the waiting list problem because they can be implemented digitally and with minimal input from a therapist [18]. Emerging evidence suggests that DMHIs are effective in improving a wide range of mental health conditions, including depression and anxiety [19,20]. Moreover, DMHIs have adopted psychotherapies, like CBT and acceptance and commitment therapy, and these have been found to be as effective as face-to-face therapies [21,22]. Due to the advantages of DMHIs, such as being more scalable, more accessible, and more cost-effective compared to face-to-face therapies [23,24], DMHIs offer the possibility to provide patients with a pre–face-to-face therapy intervention while they are on a waiting list. In addition, DMHIs can be used in a stepped care model as the frontline treatment, which steps up to more intense and advanced care if patients do not improve with the DMHIs.

However, although evidence shows that DMHIs are effective in treating depression and anxiety, it is unknown whether DMHIs are effective for patients on waiting lists. Unlike face-to-face psychotherapies that are usually delivered in a relatively standard procedure, DMHIs encompass diverse features, such as being available on multiple digital devices (website, computer, mobile apps) and offering many delivery formats (email, text message, virtual reality, games, self-help, or guided) [25-27]. It is unclear which of these features are critical for the creation and implementation of effective interventions. The typical factors undermining the effectiveness of DMHIs include low user engagement and low perceived credibility and usability; both of which are associated with poor treatment adherence and subsequently lower effectiveness [28-30]. In contrast, guided DMHIs have been found to be associated with higher user engagement [31], higher treatment adherence [32], and higher effectiveness compared to self-guided DMHIs [21,22]. In addition to user engagement, perceived credibility and usability are positively correlated with treatment outcomes [33]. Hence, it is important to investigate the perceived credibility and usability of DMHIs, as well as user engagement for patients on waiting lists in order to assess their effectiveness.

To our knowledge, there are no previous systematic reviews that synthesized the effectiveness of DMHIs for relieving symptoms of depression and anxiety for patients on waiting lists for face-to-face psychotherapy. Since depression and anxiety are the most prevalent mental health disorders globally [34], this review focused on DMHI’s impacts on alleviating depression and anxiety. This review also investigated the features, perceived credibility, and usability of DMHIs during waiting times.

## Methods

### Overview

This systematic review was conducted following the guidelines of the PRISMA (Preferred Reporting Items for Systematic Reviews and Meta-Analyses; see Multimedia Appendix 1 for PRISMA checklist) statement [35]. The study protocol was uploaded to the Open Science Framework prior to running the initial search and updates after the search have been documented.

### Search Strategy and Selection Criteria

Searches were conducted in the following 4 web-based databases: PubMed, PsycINFO, Cochrane, and Web of Science. Backward and forward citation searching was manually conducted using the Web of Science. The search was carried out in June 2024. Studies published before June 6, 2024, and that met the inclusion criteria were included in the review.

The search terms used were a combination of the population (ie, patients on the waiting lists for psychotherapy), treatment (ie, intervention), and outcome (ie, depression or anxiety, or both). The detailed search terms for each domain are shown in Table 1. In addition, the language was limited to English, and the publication type was limited to peer-reviewed publications in scientific journals with full-text access.

To fulfill the objectives of this review, the inclusion criteria were (1) randomized controlled trials (RCTs), or clinical trials, or pilot studies, or feasibility studies (2) of any kind of digital intervention (3) for individuals on waiting lists (4) for psychotherapy (5) with clear psychological outcome measurements that included depressive or anxiety symptoms.

**Table 1:** Search terms.

Concept	Search terms^a^
Concept 1: population	“before psychotherapy” OR “before therapy” OR “pretreatment” OR “pretherapy” OR “before outpatient psychotherapy” OR “waiting time” OR “waiting list”
Concept 2: intervention	“digital intervention” OR “digital mental health” OR “DMH” OR “eMental health” OR “mobile mental health” OR “mobile psychiatry” OR “technology” OR “online” OR “mobile” OR “phone” OR “app” OR “web” OR “internet” OR “computer*” OR “ehealth” OR “mhealth” OR “guided” OR “self-help”
Concept 3: outcome	“depress*” OR “anxiety” OR “MDD” OR “anxious” OR “GAD” OR “generalized anxiety disorder”

^a^Search terms are based on the inclusion criteria (see eligibility criteria), targeting the population, intervention, outcome, and study type. The search terms were combinations of the keywords listed under categories 1, 2, and 3.

### Screening and Data Extraction

Three of the coauthors (SH, GL, and TJN) independently completed the title and abstract screening of the retrieved study records, as well as the full-text reviews of the eligible studies. The web-based platform Covidence (SaaS Enterprise) was used in managing the screening process. The corresponding author resolved any screening disparities.

Data were extracted through Covidence following the same template for each study across three domains: (1) study characteristics (ie, study title, authors, publication year, country, study type, health care settings, recruitment, sample size and demographics, and inclusion and exclusion criteria); (2) intervention characteristics (ie, therapy type, components, duration, and outcome measurements); and (3) study results (ie, primary outcome: the reduction in depression and anxiety symptoms, secondary outcome: user engagement, intervention credibility, and usability).

### Data Synthesis

The extracted data were first synthesized by the study characteristics and the features of interventions including sample size, recruitment methods, types of psychotherapy adapted, delivery formats, and outcome measures. Next, we synthesized the effectiveness of the digital intervention by analyzing the reduction of depression and anxiety symptoms, separately for different study types (ie, RCTs and non-RCTs). Finally, reports about user engagement and perceived credibility and usability were synthesized.

## Results

### Overview

**Figure 1:**
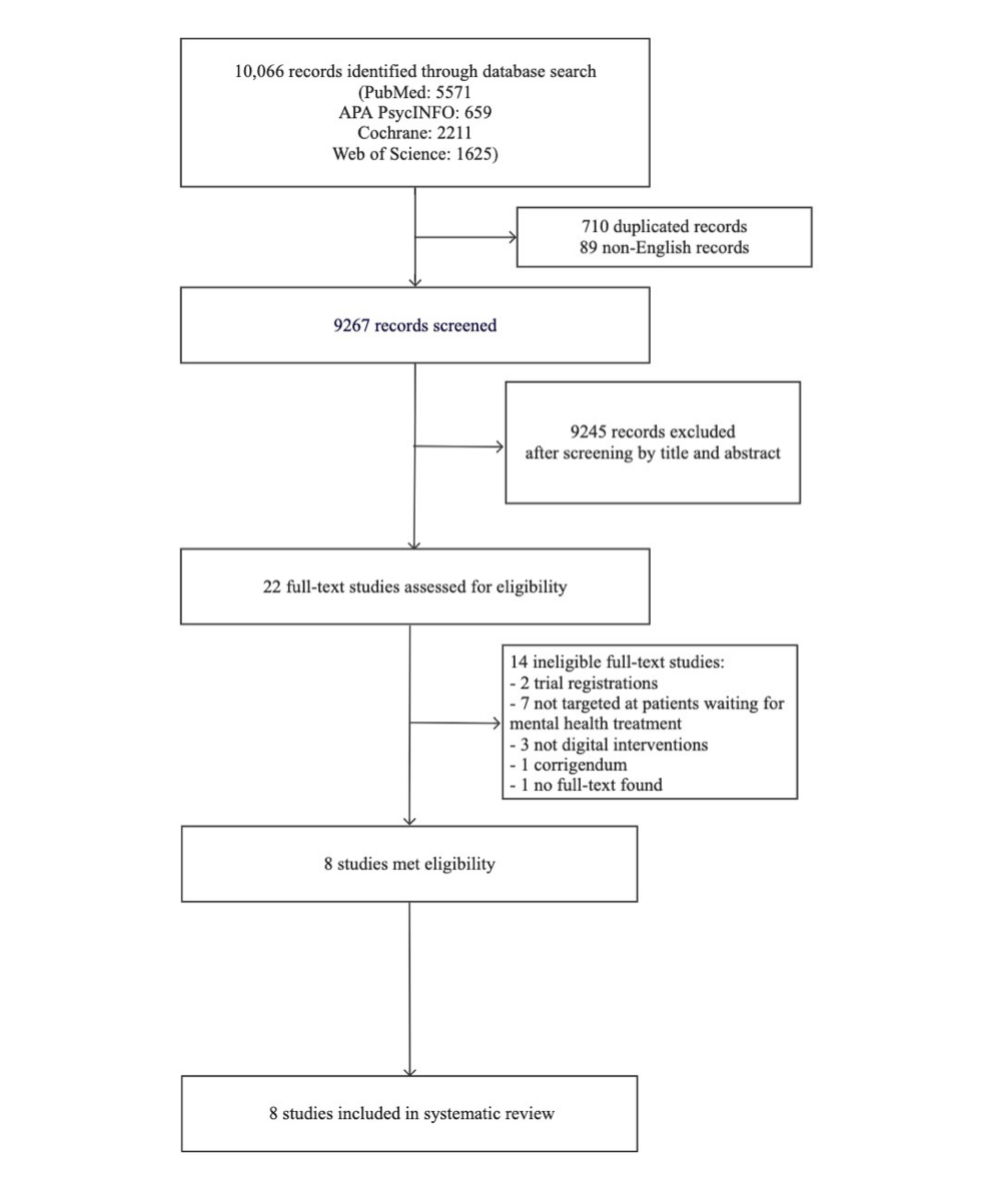
PRISMA flow diagram outlining data set and study identification. PRISMA: Preferred Reporting Items for Systematic Reviews and Meta-Analyses.

Of the 10,066 study records retrieved from the databases, and after removing duplicates and non-English studies, a total of 9267 unique titles and abstracts were screened. Following the screening, a final 22 studies remained for full-text screening (Figure 1). After scanning the full texts, 8 studies met eligibility criteria and were included in the review.

### Study Characteristics

The study characteristics are shown in Table 2. In all the studies, the participants were informed about the digital intervention by their health care service providers and were recruited if they were interested in participating and passed screening. Five studies used an RCT design, two studies were feasibility studies, and 1 study was a nonrandomized experimental study. Studies by Kenter et al [11] and Kolovos et al [36] used the same data collection for analysis and were therefore discussed together in the current review.

**Table 2:** Study characteristics.

Reference, study type, and location	Setting	Recruitment	Sample size	Demographics	Inclusion and exclusion criteria
Krämer et al (2021) [37], RCT^a^, Germany	Outpatient clinics	Individuals were informed about this study and provided with a screening questionnaire.	136	Age: mean 36.3 (SD 11.9); 78% female; 58% in a relationship	Included depressive patients on the waiting lists (age 18 years and older; CES-D^b^>22; BSI^c^ item 9≤1). Excluded patients indicating suicidal ideation at baseline.
Villemaire-Krajden and Myhr (2019) [38], RCT, Canada	A university clinical teaching unit in a tertiary care hospital	Participants were recruited from referrals for CBT^d^ and instructions were sent by emails.	67	Age: mean 38.21 (SD 12.71); 67% female; 43% in a relationship	Unspecified, but included outpatients with all types of depressive disorders, anxiety, bipolar, insomnia, psychosis, adjustment disorder, personality disorders, and autism spectrum disorder.
Kenter et al (2016) [11], RCT, The Netherlands	Outpatient clinics	Recruited patients after their registration for regular face-to-face treatments. Screened by telephone.	269	Age: mean 38 (SD 11.4); 53.9% female; 24.2% in a relationship	Included outpatients waiting for face-to-face treatment at clinics with major depressive disorder diagnosed by *DSM-IV*^e^ (age 18 years and older, waiting time >8 weeks). Excluded patients with suicidal ideation and antidepressant medication.
Kolovos et al (2016) [36], RCT, The Netherlands	Outpatient clinics	Recruited patients after their registration for regular face-to-face treatments. Screened by telephone.	269	Age: mean 38 (SD 11.4); 53.9% female; 24.2% in a relationship	Included outpatients waiting for face-to-face treatment at clinics with major depressive disorder diagnosed by *DSM-IV* (age 18 years and older, waiting time >8 weeks). Excluded patients with suicidal ideation and antidepressant medication.
Twomey et al (2014) [39], RCT, Ireland	A public health care provider in Dublin	Participants were invited by telephone, and instructions were sent by email.	149	Age: mean 35.3 (SD 10.3); 73.8% female; 41.6% in a relationship	Included adults on waiting lists with symptoms of depression, anxiety, or stress (as shown by their initial referral). Excluded those with psychosis or cognitive impairment.
Hentati et al (2022) [40], feasibility study, Sweden	A routine psychiatric care unit	Participants were informed by the health care personnel at the psychiatric care unit and self-registered digitally.	12	Age: mean 34.3 (SD 11.1); 50% female; 75% in a relationship	Included patients with symptoms of depression or anxiety on the waiting list (age 18 years and older; PHQ-9^f^ or GAD-7^g^≥5; MADRS-S^h^ item 9<4). Excluded patients with severe suicidal ideation, and psychiatric or somatic difficulties.
Duffy et al (2019) [41], feasibility study, United Kingdom	Outpatient clinics	Participants were informed of the study through their clinician and discussed their participation with clinicians during the assessment appointment.	123	Age: 28% between 17 and 24, 46% between 25 and 44, 26% between 45 and 64, and 1% between 65 and 80; 69% female	Included clients with severe presentations of anxiety or depression, or both, requiring high-intensity treatment. Excluded clients requiring low-intensity treatment and those with substance abuse.
Whitfield et al (2006) [42], nonrandomized experimental study, United Kingdom	Outpatient clinics	Referred participants were invited to a screening appointment.	20	Age: mean 38.05 (SD 12.98); 45% female	Included patients with problems of depression and depression with anxiety (aged between 16 and 65, BDI-II^i^<2). Excluded patients with current active suicidal intent and psychosis.

^a^RCT: randomized control trial.

^b^CES-D: Center for Epidemiologic Studies Depression Scale [43].

^c^BSI: brief symptom inventory [44].

^d^CBT: cognitive behavioral therapy.

^e^*DSM-IV: Diagnostic and Statistical Manual of Mental Disorders* (4th Edition).

^f^PHQ-9: Patient Health Questionnaire-9 [45].

^g^GAD-7: Generalized Anxiety Disorder-7 [46].

^h^MADRS-S: Montgomery-Åsberg Depression Rating Scale - Self Assessment [47].

^i^BDI-II: Beck Depression Inventory Version II [48].

Among the 5 RCTs, only the intervention group in Krämer et al [37] reported a significant decrease in depressive symptoms, as well as improvements in psychological symptoms and quality of life, compared to the control group. In the other 4 RCTs, 3 RCTs provided the control groups with a self-help book and did not find the internet-based interventions more effective in reducing depression and anxiety symptoms than the control groups, and 1 RCT did not provide any intervention for the control group [11,36,38,39]. The 3 non-RCT studies, which did not have a control group, reported mixed findings. Two studies found that the effectiveness of the digital interventions was beneficial and reduced depression and anxiety symptoms during the waiting time and at follow-up [41,42]; however, after the intervention in the remaining study, only a few participants reached the threshold for clinical improvement in their symptoms of depression or anxiety [40].

### Quality Assessment

We used the RoB 2 tool (a revised Cochrane risk of bias tool for randomized trials) to assess the risk of bias of the included RCTs. The RoB 2 evaluates five domains, including the randomization process, deviations from intended interventions (effect of assignment to intervention), missing outcome data, measurement of the outcome, and selection of the reported result [49]. In addition, the ROBINS-I (Risk of Bias in Nonrandomized Studies-of Interventions) tool by Cochrane was used to assess the risk of bias of the included non-RCTs. The ROBINS-I evaluates seven domains including confounding, selection of participants, classification of interventions, deviations from intended interventions, missing data, measurement of the outcomes, and selection of the reported results [50].

Out of the 5 included RCTs, 4 RCTs had low risk and 1 RCT had some concern with regard to the randomization process (due to significant baseline differences between the two groups). Two studies had low risk and three studies had some concerns regarding low intervention adherence. Regarding missing data, 2 studies had low risk and 3 studies had moderate to high risk due to dropouts. All 5 studies had some concerns due to it being unclear if the assessment method was double-blinded or not. Finally, all studies had a low risk of bias regarding the selection of the reported result. A detailed quality assessment for each study is presented in Table S1 in Multimedia Appendix 2.

The 3 included non-RCTs all had moderate risks. A common serious risk factor among all studies was the consideration of confounders. None of the studies reported potential confounders, which contributed to the potential bias of the study results. In addition, intervention adherence was a concern in all studies. Due to the moderate to high dropout rates, the handling of the missing values was problematic in two of the three studies. Finally, similar to RCTs, the 3 non-RCTs also only included self-reported outcome measurements, and it was unclear if outcome assessors were aware of the intervention received by study participants. A detailed quality assessment for each study is presented in Table S2 in Multimedia Appendix 2.

### Randomized Controlled Trials

The intervention characteristics of RCTs are shown in Tables 3 and 4. Krämer et al [37] found that the “GET.ON Mood Enhancer” that they used was effective in reducing depressive symptoms. The intervention group improved significantly after the intervention compared to the control group (*F*_2,121.5_=3.91; *P*<.05; see mean, SD, and between-group effect size in Table 5). The control group remained on the waiting list and did not receive any intervention during the study period. These differences were also maintained at the 5-month follow-up, where the depressive symptoms were significantly reduced in the intervention group compared to the control group. In addition, Krämer et al [37] reported a significant difference in the decrease of both psychological symptoms (*F*_2,119.4_=4.37; *P*<.05) and mental health quality of life (*F*_2,119.7_=3.36; *P*<.05), indicating that the intervention group improved more than controls.

**Table 3:** Intervention characteristics of RCT^a^ studies.

Reference	Therapy	Delivery formats	Components	Duration and frequency
Krämer et al (2021) [37]	CBT^b^	Internet-based platform “GET.ON Mood Enhancer”	Six consecutive modules of 45 minutes, written semistandardized feedback by an e-coach (trained psychologist), homework assignments, a digital mood diary, and an optional text message coach	7 weeks
Villemaire-Krajden and Myhr (2019) [38]	CBT	Internet-based website “Good Days Ahead” accessible via computer	Nine lessons of 30 minutes, explanations for the CBT model and its application, vignettes presenting case examples, quizzes, exercises, introduction to a few behavioral techniques such as activity monitoring and scheduling	9 weekly lessons
Kenter et al (2016) [11]	PST^c^	Internet-based platform, email reminders if not finishing a session	Five sessions with structured homework assignments, adopting a structured 6-step approach: identifying the problem, finding solutions, selecting one solution, creating a plan to solve the problem with this solution, executing the plan, and evaluating the plan, plus web-based feedback by a coach	5 weekly sessions
Kolovos et al (2016) [36]	PST	Internet-based platform, email reminders if not finishing a session	Five sessions with structured homework assignments, adopting a structured 6-step approach: identifying the problem, finding solutions, selecting one solution, creating a plan to solve the problem with this solution, executing the plan, and evaluating the plan, plus web-based feedback by a coach	5 weekly sessions
Twomey et al (2014) [39]	CBT	Internet-based platform “MoodGYM,” weekly automated email reminders	A brief introductory session and five 20- to 40-minute sessions, containing written information, animations, human-centered exercises, and quizzes	32 days

^a^RCT: randomized controlled trial.

^b^CBT: cognitive behavioral therapy.

^c^PST: problem-solving therapy.

**Table 4:** Control group and outcome measures of RCT^a^ studies.

Reference	Control group	Measurement		
		Depression	Anxiety	Others
Krämer et al (2021) [37]	Access to treatment as usual was not restricted, participants may be seeing a general practitioner, psychiatrist, psychotherapist, using self-help, or other inpatient or outpatient practitioner	PHQ-9^b^	Not measured	Psychological symptoms (BSI^c^), quality of life—mental and physical (SF-12^d^), need and motivation for psychotherapy, attitude toward F2F psychotherapy (ATSPPH^e^), attitude toward web-based interventions (adapted ATSPPH), negative effects (INEP^f^), outpatient psychotherapy history
Villemaire-Krajden and Myhr (2019) [38]	Provided with a workbook	BDI-II^g^	BAI^h^	Subjective well-being, problem or symptoms, life functioning difficulties, and risk or harm to self or others (CORE-OM^i^)
Kenter et al (2016) [11]	Received a self-help book without any guidance or further instructions	CES-D^j^	HADS-A^k^	Insomnia (ISI^l^), self-rated health (EQ-5D [51]), perceived control (PMS^m^)
Kolovos et al (2016) [36]	Received a self-help book without any guidance or further instructions	CES-D	Not measured	Response to treatment and quality of life (EQ-5D)
Twomey et al (2014) [39]	No intervention at all	DASS^n^-Depression	DASS-Anxiety	DASS-Stress, work and social functioning (WSAS^o^)

^a^RCT: randomized controlled trial.

^b^PHQ-9: Patient Health Questionnaire-9 [45].

^c^BSI: brief symptom inventory [44].

^d^SF-12: 12-Item Short-Form Health Survey [52].

^e^ATSPPH: Attitude Toward Seeking Professional Psychological Help Scale [53].

^f^INEP: inventory for the assessment of negative effects of psychotherapy [54].

^g^BDI-II: Beck Depression Inventory Version II [48].

^h^BAI: Beck Anxiety Inventory [55].

^i^CORE-OM: Clinical Outcomes in Routine Evaluation Outcome Measure [56].

^j^CES-D: Center for Epidemiologic Studies Depression Scale [43].

^k^HADS-A: Hospital Anxiety and Depression Scale Anxiety subscale [57].

^l^ISI: Insomnia Severity Index questionnaire [58].

^m^PMS: Pearlin Mastery Scale [59].

^n^DASS-21: Depression, Anxiety, and Stress Scale-21 [60].

^o^WSAS: Work and Social Adjustment Scale [61].

**Table 5:** Statistical results of reviewed studies.

Reference	Depression symptom, mean (SD)			Anxiety symptom, mean (SD)			Between-group effect size: interaction effect, time × group
	Intervention	Control	Intervention		Control		
Krämer et al (2021) [37]	Before treatment (ITT^a^): 14.6 (0.5); posttreatment (ITT): 10.5 (0.7); five-month follow-up (ITT): 9.9 (0.7)	Before treatment (ITT): 15.7 (0.5); posttreatment (ITT): 13.3 (0.6); five-month follow-up (ITT): 12.7 (0.7)	Not measured		Not measured	Posttreatment: *P*=.004, Cohen *d*=0.55; five month follow up: *P*=.005, Cohen *d*=0.52	
Villemaire-Krajden and Myhr (2019) [38]	Before treatment: 25.50 (11.94); posttreatment: 24.14 (12.50)	Before treatment: 29.27 (11.85); posttreatment: 28.59 (14.70)	Before treatment: 22.33 (14.38); posttreatment: 20.00 (13.42)		Before treatment: 19.86 (10.61); posttreatment: 18.32 (11.65)	*P*>.05, partial eta squared=0 for both depressive and anxiety symptoms	
Kenter et al (2016) [11] and Kolovos et al (2016) [36]	Before treatment: 37.0 (11.6); posttreatment: 27.0 (15.1)	Before treatment: 35.2 (12.1); posttreatment: 25.9 (14.9)	Before treatment: 12.4 (3.9); posttreatment: 10.5 (5.4)		Before treatment: 12.6 (4.6); posttreatment: 10.0 (5.5)	Depression: Cohen *d*=0.07; Anxiety: Cohen *d*=0.09	
Twomey et al (2014) [39]	Before treatment: 18.79 (11.46); posttreatment: 14.29 (10.62)	Before treatment: 16.26 (9.71); posttreatment: 16.26 (10.52)	Before treatment: 12.93 (8.06); posttreatment: 11.71 (8.91)		Before treatment: 10.32 (7.14); posttreatment: 11.79 (9.56)	Depression: Cohen *d*=0.19; Anxiety: Cohen *d*=0.01	
Hentati et al (2022) [40]	No data provided	N/A^b^	No data provided		N/A	N/A	
Duffy et al (2019) [41]	Before-posttreatment: decrease 3.6 points	N/A	Before-posttreatment: decrease 3.2 points		N/A	N/A	
Whitfield et al (2006) [42]	Before treatment (ITT): 28.15 (11.41); posttreatment (ITT): 20.00 (10.41); three-month follow-up (ITT): 18.95 (10.41)	N/A	Before treatment (ITT): 20.30 (11.23); posttreatment (ITT): 14.55 (7.82); three-month follow-up (ITT): 9.90 (8.47)		N/A	N/A	

^a^ITT: intention-to-treat.

^b^Not applicable.

Villemaire-Krajden and Myhr [38] investigated the effectiveness of using computerized CBT to decrease symptoms of distress in outpatients during a waiting period for services. They did not find significant interactions between the intervention and control group, with a very small between-group effect size (Table 5). This indicates that compared to the control group, the intervention group did not score significantly better with regard to well-being, symptom severity, functioning, or motivation for CBT. In addition, depressive symptoms, anxiety symptoms, and life functioning difficulties were not found to have changed significantly between groups from baseline to the postintervention assessment. However, for both groups, the well-being (measured by Clinical Outcomes in Routine Evaluation Outcome Measure) of the participants significantly increased (*F*_1,37_=6.31; *P*<.05), and the symptoms significantly decreased (*F*_1,37_=8.74; *P*<.05) over time.

Both Kenter et al [11] and Kolovos et al [36] investigated the effectiveness of a web-based PST, self-help intervention in reducing depression symptoms. The intervention examined by Kenter et al [11] was effective in reducing depression and anxiety symptoms, but in comparison with the control group (who were provided with a self-help book), it was not more effective. They used intention-to-treat (ITT) analysis and found a significant reduction in depressive symptoms in both the intervention group (see Table 5, within-group effect size: Cohen *d*=0.75) and the control group (within-group effect size: Cohen *d*=0.69) from baseline to posttreatment. Despite the moderate within-group effect size for both groups, the between-group effect size was small (Table 5). The between-group difference posttreatment was not significant in the regression analysis. Similarly, for anxiety, Kenter et al [11] reported moderate within-group effect sizes (intervention group: Cohen *d*=0.41; control group: Cohen *d*=0.52) and small between-group effect size (Table 5), and they found no significant difference between the intervention group and the control group. The clinical outcomes in Kolovos et al [36] were the same as Kenter et al [11] but Kolovos et al [36] focused on estimating the cost-effectiveness of the intervention in comparison with enhanced usual care. Kolovos et al [36] found that from a societal perspective, the intervention was not cost-effective in comparison with the enhanced usual care.

Twomey et al [39] examined the effectiveness of a self-help CBT intervention (“MoodGYM”) on reducing general psychological distress, stress, depression, anxiety, and impaired daily functioning symptoms. Twomey et al [39] found symptom improvement for the treated individuals with a within-group effect size of Cohen *d*=0.4 for depression and Cohen *d*=0.14 for anxiety. Nevertheless, similar to the studies conducted by Kenter et al [11] and Kolovos et al [36] they did not find significant differences between the intervention group and the simply waiting control group (depression: *F*_1,64_=2.99; *P*>.05; anxiety: *F*_1,64_=1.72; *P*>.05) [39]. At posttreatment, small between-group effect sizes were found for both depression and anxiety (Table 5), suggesting that “MoodGYM” was not more effective than the control condition.

### Non-RCTs

Intervention characteristics of non-RCTs are shown in Table 6. Hentati et al [40] tested the effectiveness of a self-guided PST intervention. The results showed a 16% and 22% median for symptom improvement in depression and anxiety, respectively, from screening to posttreatment. However, from pretreatment to posttreatment, the improvement in depression was reduced by 3%, while anxiety symptoms were reduced by 3%. Hentati et al [40] concluded that there was insufficient evidence for the effectiveness of this digital intervention.

**Table 6:** Intervention characteristics of non-RCT^a^ studies.

Reference	Therapy	Delivery formats	Components	Duration and frequency	Measurement		
					Depression	Anxiety	Others
Hentati et al (2022) [40]	CBT^b^	Internet-based platform accessible via computer and mobile devices	Psychoeducational texts, treatment rationale, examples of problems and suggestions of solutions, illustrative pictures, instructions, and problem-solving exercises	4 weeks	PHQ-9^c^	GAD-7^d^	Treatment credibility (CEQ^e^), usability (SUS^f^), behavioral engagement, suicidal ideation (MADRS-S^g^), negative effects (NEQ^h^), poststudy questionnaire on user experience
Duffy et al (2019) [41]	CBT	Internet-based platform “SilverCloud”	Eight modules including tools such as self-monitoring and thought recording, behavioral activation, cognitive restructuring, and challenging core beliefs	47 days, one review every 10 to 12 days.	PHQ-9	GAD-7	Work and social functioning (WSAS^i^)
Whitfield et al (2006) [42]	CBT	Computerized CD Rom	Six sessions of 45-60 minutes, including text, cartoon illustrations, animations, human-centered questions, sound, and video, as well as the offer of short support sessions by a self-help support psychiatric nurse	6 weekly sessions	BDI-II^j^	BAI^k^	Hopelessness (BHS^l^), social adaptation self-evaluation (SASS^m^)

^a^RCT: randomized controlled trial.

^b^CBT: cognitive behavioral therapy.

^c^PHQ-9: Patient Health Questionnaire-9 [45].

^d^GAD-7: Generalized Anxiety Disorder-7 [46].

^e^CEQ: Credibility/Expectancy Questionnaire [62].

^f^SUS: System Usability Scale [63].

^g^MADRS-S: Montgomery-Åsberg Depression Rating Scale - Self Assessment [47].

^h^NEQ: Negative Effects Questionnaire [64].

^i^WSAS: Work and Social Adjustment Scale [61].

^j^BDI-II: Beck Depression Inventory Version II [48].

^k^BAI: Beck Anxiety Inventory [55].

^l^BHS: Beck Hopelessness Scale [65].

^m^SASS: Social Adaptation Self-evaluation Scale [66].

Duffy et al [41] examined the outcomes of using iCBT as a prequel for patients requiring high-intensity treatment. The results indicated that 58% exhibited reliable improvement from baseline to iCBT exit and around 20% of the sample achieved reliable recovery in advance of starting face-to-face therapy. This was demonstrated by the decrease in depression (within-group effect size: Cohen *d*=0.61) and anxiety (within-group effect size: Cohen *d*=0.69). In addition, the decrease in the WSAS score, which estimates the severity of work and social adjustment impairment, followed a similar pattern with a substantial reduction (mean 2.4, SD 8.7 points) in the severity score at iCBT exit (within-group effect size: Cohen *d*=0.31).

Finally, Whitfield et al [42] investigated the effectiveness of the compact disc CBT self-help intervention. They adopted an ITT analysis where the last observation for the participant was carried forward if data were missing due to questionnaire nonresponse. The ITT analysis could possibly reduce any potential bias by accounting for the situation of those who had dropped out of the study. Although they did not calculate the effect sizes, the mean BDI-II scores for depression symptoms significantly decreased (*t*_19_=4.91; *P*<.001), and the mean BAI score for anxiety symptoms significantly decreased as well (*t*_19_=2.51; *P*=.02; Table 5). Furthermore, Whitfield et al [42] found the effectiveness of the intervention persisted at the 3-month follow-up.

### Summary

To summarize, there is some evidence of the effectiveness of digital interventions to improve depression and anxiety symptoms in individuals waiting for psychotherapy. Seven of the eight studies reported digital interventions that led to a reduction in depressive and anxiety symptoms, with, on average, a moderate within-group effect size. However, only one study found that the waiting list intervention was effective in comparison with the control group. Moreover, due to the small between-group effect sizes in four of the five RCTs and the small sample sizes in the non-RCTs, it is difficult to conclude that the waiting list interventions were effective.

The secondary outcomes are shown in Table S3 in Multimedia Appendix 2. In brief, the reviewed studies showed that the user engagement of the self-guided digital interventions was low, but the guided digital interventions appeared more engaging compared to the unguided ones. The low engagement and high dropout rates may have contributed to the interventions being less effective. The interventions were generally perceived as credible and useful, with moderate to high user satisfaction, suggesting that digital interventions have the potential to be adapted as a pretreatment for patients on waiting lists.

## Discussion

### Principal Findings

This systematic review identified 8 studies of digital interventions aimed at reducing the depression and anxiety symptoms of patients waiting for face-to-face psychotherapy. We found that waiting list interventions were effective in reducing symptoms of depression and anxiety, but not more effective compared to simply waiting for treatment or control groups who used a self-help book. Although the digital interventions were perceived as credible and useful, low user engagement was a major concern for treatment adherence and effectiveness.

### Effectiveness and Features of the Waiting List Digital Interventions

We found that the DMHIs implemented with patients on waiting lists reduced depression and anxiety symptoms with moderate effect sizes. These effect sizes were similar to those of prior meta-analyses on the effectiveness of DMHIs in general by Firth et al [22] and Moshe et al [28]. Nevertheless, this review did not find significant differences between the intervention group and the control group. Several factors might have reduced symptoms in the control groups. First, in the RCTs conducted by Kenter et al [11] and Kolovos et al [36], the control group was provided with a self-help book instead of merely waiting; the aim was to increase the participation rate. Even with small effect sizes, previous studies have shown that self-help books were effective in reducing depression symptoms [8]. In addition, some spontaneous improvements were found among the waiting control group, as demonstrated in Twomey et al [39]. This is in line with previous studies that show that spontaneous improvement does occur among some patients with depression and those with anxiety [16,67].

Concerning the features of the DMHIs, there was no difference between DMHIs implemented for patients specifically on waiting lists and general DMHIs. In addition, across the reviewed 8 studies, we did not find evidence of a specific technological platform (ie, using phone vs computer) or a specific psychological therapy (ie, CBT vs PST) having better effectiveness in DMHIs, which is consistent with previous studies [8,28].

While DMHIs have the potential to provide more scalable, more accessible, and more cost-efficient treatments compared to face-to-face therapies [23,24], it is important to acknowledge that DMHIs may not be suitable to address some patients’ need for face-to-face psychotherapy, especially for those with severe symptoms [11,36]. Due to these concerns, Twomey et al [39] and Krämer et al [37] recommended that digital interventions should not be provided as a substitute for face-to-face psychotherapy but were better suited to being an additional or complementary treatment option that could bridge the waiting time. This is consistent with Cornish’s [68] stepped-care model, where patients are able to receive timely digital interventions on the same day that they seek care, balancing out the treatment intensity and resources available.

### User Engagement

As identified by earlier studies, guided interventions are more engaging compared to unguided ones [31,32], and general DMHIs implemented during the waiting time exhibited lower user engagement and treatment adherence compared to face-to-face psychotherapies [28-30]. In this review, Krämer et al [37] show that there was a lower dropout rate and higher treatment adherence compared to the other self-guided studies. This might be explained by the participants’ demographics. For example, Twomey et al [39] reported that although 85% of the males dropped out of the study, the number of females that dropped out was only 8%. In contrast, Kenter et al [11] and Kolovos et al [36] reported that 64.7% of the participants who did not complete the study were female. They also found that participants who were younger, less educated, and had lower incomes were more likely to drop out [11,36]. In addition, as Farrington et al [69] suggest, technical difficulties experienced by certain age groups (ie, older people) and incompatibility between different digital device systems may also have been barriers to user engagement.

Another factor for low user engagement might be related to how the digital intervention was promoted. In all the studies reviewed, the interventions were presented to the participants as an additional or temporary treatment rather than a replacement for the face-to-face psychotherapy they were waiting for. Promoting the interventions in this way might have lowered the expectancy of participants as regards the effect of the treatment and they may also have perceived it as less important. For instance, Hentati et al [40] reported that the participants were not convinced that the digital intervention would result in any major reduction in their symptoms although they perceived the intervention as credible. Kenter et al [11] found that the completion rate was positively associated with participants’ expectancy and perceived credibility of the intervention. Therefore, changing the way digital interventions are promoted may increase the engagement rate.

### Limitations and Future Studies

Due to the understudied nature of the topic, the first limitation of this systematic review was the restricted number of randomized control studies (n=5), which reduces the possibility of drawing a robust conclusion concerning the effectiveness of waiting list interventions. In addition, according to the quality assessment, the reviewed studies had some shared concerns about using self-report data and missing values due to dropouts. In future studies, once a sufficient number of high-quality studies with adequate sample sizes are available, conducting a meta-analysis will be crucial to provide a more comprehensive synthesis of the evidence in this area. Second, various instruments were adopted to measure depression and anxiety symptoms, posing challenges when controlling for baseline differences and when comparing the effectiveness of the studies. Moreover, the psychological outcomes were self-reported in most studies, and these might be less sensitive to symptom changes compared to a clinician’s assessment [37]. Third, this study was limited to patients with depression and those with anxiety with relatively stable conditions. Patients with severe suicidal ideation and psychosis were excluded, even though these groups are at a higher risk for symptom deterioration while waiting for psychotherapy. Ketner et al [11] reported one case of suicide in the control group, suggesting that future waiting list interventions could target individuals with less stable conditions. Additionally, when considering future studies, we recommend the use of multiple research protocols for RCTs in the field of digital interventions for patients on waiting lists that target a wider range of symptoms (ie, phobic disorders and eating disorders) [70-72]. Moreover, we recommend that researchers adopt more therapies (ie, exposure therapy) and include larger samples [73,74]. Finally, it is also important for future studies to consider the use of digital interventions in different populations. We acknowledge that this review only included English studies, which may have missed valuable studies conducted in non-English speaking countries. The reviewed studies in this study were also conducted in high-income Western countries. It is unclear whether resource-limited areas may benefit more from low-cost, web-based interventions for mental health issues.

### Conclusions

As a potential solution to the problem of prolonged waiting lists for psychotherapy, this systematic review examined the effectiveness of DMHIs in reducing anxiety and depression symptoms of patients on waiting lists for psychotherapy. Our results showed that among the RCTs, DMHIs were overall not more effective when compared with simply waiting or the control groups who used a self-help book. Among the non-RCTs, although the intervention reduced depression and anxiety symptoms, the study design with no control group made it difficult to conclude the effectiveness of the intervention. Waiting list DMHIs may prove to be an adequate treatment for some patients, but low user engagement remains a concern for treatment adherence and effectiveness. Despite patients rating DMHIs as credible and useful, it is nevertheless evident that as yet DMHIs might not fully replace face-to-face psychotherapies for all patients.

## Appendix

Multimedia Appendix 1 PRISMA (Preferred Reporting Items for Systematic Reviews and Meta-Analyses) checklist.

Multimedia Appendix 2 Quality assessments and secondary outcomes.

## Abbreviations

CBT: cognitive behavioral therapy
DMHI: digital mental health intervention
ITT: intention-to-treat
PRISMA: Preferred Reporting Items for Systematic Reviews and Meta-Analyses
PST: problem-solving therapy
RCT: randomized controlled trial
ROBINS-I: Risk of Bias in Nonrandomized Studies-of Interventions
