# Correction: Digital Mental Health Interventions for Alleviating Depression and Anxiety During Psychotherapy Waiting Lists: Systematic Review

**DOI:** 10.2196/67281

**Published:** 2024-10-16

**Authors:** Sijia Huang, Yiyue Wang, Gen Li, Brian J Hall, Thomas J Nyman

**Affiliations:** 1 Faculty of Arts and Sciences New York University Shanghai Shanghai China; 2 Center for Global Health Equity New York University Shanghai Shanghai China; 3 School of Global Public Health New York University New York, NY United States; 4 Health, Behavior and Society Johns Hopkins Bloomberg School of Public Health Baltimore, MD United States; 5 School of Psychology and Clinical Language Sciences University of Reading Reading United Kingdom

In “Digital Mental Health Interventions for Alleviating Depression and Anxiety During Psychotherapy Waiting Lists: Systematic Review” (JMIR Ment Health 2024;11:e56650) 2 errors have been noted:

In Table 3, row “Twomey et al (2014)”, column 4:

A brief introductory session and five 2- to 40-minute sessions

has been revised to:

A brief introductory session and five 20- to 40-minute sessions

In Table 6, row “Whitfield et al (2006)”, column 4:

Six sessions of 4-60 minutes

has been revised to:

Six sessions of 45-60 minutes

The correction will appear in the online version of the paper on the JMIR Publications website on October 16, 2024 together with the publication of this correction notice. Because this was made after submission to PubMed, PubMed Central, and other full-text repositories, the corrected article has also been resubmitted to those repositories.

