# Supplementary material for: Digital Mental Health Interventions for Alleviating Depression and Anxiety During Psychotherapy Waiting Lists: Systematic Review
Source: JMIR Ment Health. 2024 Sep 10;11:e56650. doi: 10.2196/56650 (PMC11422735; doi:10.2196/56650)
Supplement: Multimedia Appendix 2 [file mental_v11i1e56650_app2.docx]

**Table S1**

*Quality assessments of RCT studies using the RoB 2 tool.*

| Reference | Randomization process | Deviations from Intended Interventions | Missing Outcome Data | Measurement of the Outcome | Selection of the Reported Result | Overall Risk of Bias |
| --- | --- | --- | --- | --- | --- | --- |
| Krämer et al., 2021 | Low risk (allocation sequence was random and two groups matched at baseline) | Low risk (experimental group received immediate intervention, while the control group received intervention after follow-up; adherence to the intervention was good) | Some concerns (dropout rates were 25% and 40% at follow-up) | Some concerns (self-reported data, unclear if outcome assessors were aware of the intervention received by study participants) | Low risk (the study was pre-registered and reported all pre-specified outcomes) | Low risk to some concerns |
| Villemaire-Krajden et al., 2019 | Low risk (allocation sequence was random and two groups matched, except for age, at baseline) | Low risk (experimental group received immediate intervention, while the control group received a CBT workbook; adherence to the intervention was good) | Some concerns (unclear about how missing data was handled) | Some concerns (self-reported data, unclear if outcome assessors were aware of the intervention received by study participants) | Low risk (the study reported all pre-specified outcomes) | Low risk to some concerns |
| Kenter et al., 2016 & Kolovos et al., 2016 | Low risk (allocation sequence was random and two groups matched at baseline) | Some concerns (adherence to the intervention was low) | Low risk (multiple imputation was used to handle missing data) | Some concerns (self-reported data, unclear if outcome assessors were aware of the intervention received by study participants) | Low risk (the study was pre-registered and reported all pre-specified outcomes) | Low risk to some concerns |
| Twomey et al., 2014 | Some concerns (significant baseline differences between two groups) | Some concerns (adherence to the intervention was low) | High risk (high dropout rates, missing data mishandled) | Some concerns (self-reported data, unclear if outcome assessors were aware of the intervention received by study participants) | Low risk (the study reported all pre-specified outcomes) | Some concerns to high risk |

**Table S2**

*Quality assessments of non-RCT studies using the ROBINS-I tool.*

| Reference | Confounding | Selection of Participants | Classification of interventions | Deviations from Intended Interventions | Missing Outcome Data | Measurement of the Outcome | Selection of the Reported Result | Overall Risk of Bias |
| --- | --- | --- | --- | --- | --- | --- | --- | --- |
| Hentati et al., 2022 | Serious risk (no mention of potential confounders, such as severity of symptoms, comorbidity, seeking and receiving other treatment) | Low risk (participants were recruited from a routine psychiatric care unit) | Low risk (intervention group was clearly defined, no indication that the classification of intervention status could have been affected by knowledge of the outcome or risk of the outcome) | Moderate risk (no report of adherence rate) | Serious risk (high dropout rates, missing data mishandled) | Moderate risk (self-reported data, unclear if outcome assessors were aware of the intervention received by study participants) | Low risk (the study reported all pre-specified outcomes) | Moderate risk |
| Duffy et al., 2019 | Serious risk (no mention of potential confounders, such as severity of symptoms, comorbidity, seeking and receiving other treatment) | Low risk (participants were recruited from outpatient clinics and assessments were done by clinicians) | Low risk (intervention group was clearly defined, no indication that the classification of intervention status could have been affected by knowledge of the outcome or risk of the outcome) | Moderate risk (completion rate for iCBT was low) | Serious risk (high dropout rates, unclear about missing data) | Moderate risk (self-reported data, unclear if outcome assessors were aware of the intervention received by study participants) | Low risk (the study reported all pre-specified outcomes) | Moderate risk |
| Whitfield et al., 2006 | Serious risk (no mention of potential confounders, such as severity of symptoms, comorbidity, seeking and receiving other treatment) | Low risk (participants were recruited from outpatient clinics and received screening) | Low risk (intervention group was clearly defined, no indication that the classification of intervention status could have been affected by knowledge of the outcome or risk of the outcome) | Moderate risk (only 26% attended at least one section) | Low risk (the study was transparent about missing data and used an intention-to-treat analysis) | Moderate risk (self-reported data, unclear if outcome assessors were aware of the intervention received by study participants) | Low risk (the study reported all pre-specified outcomes) | Low to moderate risk |

**Table S3**

*Intervention secondary outcomes: user engagement, intervention credibility and usability.*

| Reference | User Engagement | Intervention Credibility and Usability |
| --- | --- | --- |
|  |  |  |
| Hentati et al., 2022 | 15% of the participants have never used the intervention, and 58% did not complete any evaluation of a problem-solving attempt. | For credibility, the intervention received a 33.4 mean score out of 50, and 60% of individuals considered the usability of the intervention to be “good.” |
| Krämer et al., 2021 | On average, participants adhered to four out of six modules, and 52% of them completed at least five modules. The overall dropout rate was 20%. | The study did not have results about intervention credibility and usability. |
| Duffy et al., 2019 | N/A | N/A |
| Villemaire-Krajden et al., 2019 | 61% participants in the intervention group completed all 9 lessons, and 29% in the control group completed the entire workbook. | Half of the participants found the intervention either very or extremely useful, especially rated positive on the content. Some favored the format or structure, and the independence or autonomous use of the intervention. |
| Kenter et al., 2016 | The study attrition rate was 32% and only 13% of participants completed the full five sessions. | Participants rated their satisfaction of the intervention at a mean score of 7 out of 10, only 8% perceived that the intervention was not useful. |
| Kolovos et al., 2016 | The study attrition rate was 32% and only 13% of participants completed the full five sessions. | Participants rated their satisfaction of the intervention at a mean score of 7 out of 10, only 8% perceived that the intervention was not useful. |
| Twomey et al., 2014 | Dropout rate was 56% and the completion rate was low: only 27% completed all five sessions, and 46% completed fewer than three. | 63% participants were satisfied with MoodGYM, 68% would recommend it to others, 54% believed MoodGym had benefited them, and 44% agreed that their understanding of CBT had improved because of MoodGYM. |
| Whitfield et al., 2006 | Participation rate was 26% which was low. 30% of the participants (six individuals) dropped out, among which, five dropped out after the first week and one dropped out after the third week. | 60% users reported that the intervention had been useful to the extent of “a lot”, and 27% said that the intervention had improved their mood “a lot”. All users rated the experience positively and would recommend it to others. Only one user (7%) reported that the program was “not at all” easy to understand. |

*Note*. In Duffy et al., the therapeutic alliance was evaluated as a collective processing including both iCBT and the following high-intensity treatment (face-to-face).[40] As such, it is difficult to distinguish the user engagement and intervention credibility and usability specifically during the iCBT intervention.
